# Latent profiles of death anxiety among young adults: associations with self-esteem, security, and perceived social support

**DOI:** 10.3389/fpsyt.2025.1594720

**Published:** 2025-09-24

**Authors:** Jingxian Yu, Mingjie Wu, Yongqi Liang, Huan Peng, Na Li, Hanjiao Liu

**Affiliations:** ^1^ Shenzhen Clinical College of Integrated Chinese and Western Medicine, Guangzhou University of Chinese Medicine, Shenzhen, China; ^2^ The Seventh Clinical Medical College of Guangzhou University of Chinese Medicine, Shenzhen, China; ^3^ Fujian University of Traditional Chinese Medicine, Fuzhou, China

**Keywords:** death anxiety, self-esteem, perceived social support, security, latent profile analysis, young adults

## Abstract

**Introduction:**

Death anxiety is a critical mental-health concern among young adults; however, its heterogeneity and underlying psychological mechanisms remain understudied. This study aimed to identify latent profiles of death anxiety in Chinese youth and examine the predictive roles of self-esteem, perceived social support, and security.

**Methods:**

We conducted a cross-sectional survey of 623 young adults (*mean age* = 23.62 years, *SD* = 3.61) aged 18–35 years in mainland China. Latent profile analysis (LPA) was conducted to classify death anxiety subgroups based on responses to the Templer Death Anxiety Scale (C-T-DAS). Self-esteem, perceived social support, and sense of security were assessed using validated scales. Multinomial logistic regression and ANOVA were used to explore predictors and group differences.

**Results:**

Three latent death anxiety profiles emerged, High Death Anxiety (56.2%), Moderate Cognition and Low Death Anxiety (8.8%), and Low Cognition and Moderate Death Anxiety (35%). Higher self-esteem (*β* = -0.46, *p* <.001), social support (*β* = -1.12, *P = .*004), and security (*β* = -2.87, *P* <.001) significantly predicted lower death anxiety. The high death anxiety group exhibited the lowest psychological resource scores. Older age (30–35 years) and recent acute illness recovery were associated with higher death anxiety risk (*OR* = 0.28, 95% *CI* [0.09, 0.93]). Security showed the strongest inverse association with DA (*F =* 50.72, *P* <.001), particularly in the interpersonal and controllability dimensions.

**Conclusion:**

Death anxiety among young adults is heterogeneous, influenced by distinct psychological profiles and demographic factors. Interventions should prioritize enhancing self-esteem, social support networks, and security to mitigate death anxiety, especially in high-risk subgroups. Future research should employ longitudinal designs and cross-cultural samples to validate causal pathways and refine targeted strategies.

## 1 Introduction

Against accelerated globalization and social uncertainty, Chinese youth (18–35) face academic, professional, and social pressures that threaten mental health, while modern media repeatedly expose them to death-related images (e.g., COVID-19 mortality) (1–6). At this identity-forming stage, such exposure heightens death anxiety (DA), which—if unaddressed—can precipitate social withdrawal, loss of meaning, and depressive mood (7–10).

Death anxiety is a fundamental psychological phenomenon characterized by fear and worry about one’s own death or that of others, typically manifesting as intense distress and emotional reactions related to dying, uncertainty about what happens after death, and similar themes (11). It has been identified as a significant factor associated with a variety of mental health problems, including generalized anxiety disorder, depression, obsessive-compulsive disorder, panic disorder, and schizophrenia (10, 12, 13). Additionally, death anxiety is thought to influence the development of several somatic symptom disorders, such as muscle dysmorphia and other medically unexplained symptoms (13, 14).

Empirical studies have shown that high death anxiety may affect an individual’s health behaviors, such as decreased sleep quality and loss of appetite, and that these behavioral changes may further impair somatic health and exacerbate death anxiety (14, 15). Certain occupational groups, such as doctors, nurses, and embalmers, are subject to greater psychological stress due to occupational characteristics that require frequent exposure to death scenes. This stress can exacerbate their death anxiety, which in turn negatively affects their professional identity, self-efficacy, and self-confidence. This not only reduces the quality of their response to the needs of the patient or dying person but may also cause secondary victimization of the patient or bereaved person in their care (16, 17).

Despite its importance, the existing literature on death anxiety has primarily focused on older adults, terminally ill patients, and healthcare professionals (18–20). Limited attention has been given specifically to young adults, who may experience unique vulnerabilities due to the critical developmental tasks they face, including identity formation and value establishment (21). Furthermore, previous studies have predominantly adopted variable-centered approaches, analyzing the linear relationship between death anxiety and single variables. This approach often overlooks the heterogeneity within youth populations, neglecting the fact that different individuals may develop differentiated anxiety patterns through unique combinations of psychological profiles, such as varying levels of self-esteem, perceived social support, and security (22).

Previous research has explored a variety of psychological factors that influence death anxiety. In the context of Chinese youth facing conflicting traditional and modern death-related beliefs, Terror Management Theory (TMT) provides an important theoretical framework, suggesting that individuals alleviate death anxiety by reinforcing cultural worldviews, maintaining self-esteem, and building close relationships, collectively referred to as the “Death Anxiety-Buffer System” (DABS) (23). However, young people in Mainland China are currently undergoing a period of cultural worldview adjustment, and the conflict between traditional and modern concepts has led to the fragility of the buffer system, which has weakened its mitigating effect on death anxiety. For example, the tension between traditional Confucian views (e.g., “without knowing life, how to know death”) and modern death-related information (e.g., media reports of epidemics) may weaken the effectiveness of these buffering mechanisms and make individuals more vulnerable to death anxiety (24). Despite the impact of the macro-cultural context on the death anxiety buffering system, individual-level factors favoring the alleviation of death anxiety still play an important role.

Therefore, rather than conducting a comprehensive assessment of death anxiety as a single factor, this study specifically focuses on identifying its heterogeneous profiles among young adults and examining how key psychological resources predict these profiles.

To address these research gaps, this study employs a person-centered approach using latent profile analysis (LPA) to explore heterogeneity in death anxiety among young adults. This method allows for the identification of distinct subgroups characterized by unique psychological profiles (25). Additionally, we investigate the predictive roles of self-esteem, perceived social support, and security, which are theoretically suggested to be crucial buffers against death anxiety (26, 27). By providing a nuanced understanding of death anxiety manifestation among youth, our findings aim to inform targeted psychological interventions and enhance mental health support specifically tailored to young adults.

Self-esteem refers to how people evaluate themselves, including the way they view themselves, the respect they have for themselves, and the appreciation of their value in specific areas (28). Terror Management Theory suggests that chasing meaning provided by cultural worldviews and maintaining self-esteem can give a sense of worth and alleviate anxiety, which can help manage death-related anxiety (29). A large body of data demonstrates that self-esteem serves as a buffering mechanism to help individuals withstand damage from death anxiety. Belmi et al.’s experiments demonstrated that death salience motivates people to increase their sense of security and self-evaluation to manage death anxiety by, for example, seeking psychological security and enhancing self-esteem (27). At the same time, Zhang et al. observed that by reviewing successful experiences and searching for the meaning of life, individuals can gain affirmation of their value and significance, thus reducing anxiety caused by the inevitability of death (30).

Social support is defined as the feeling that a person is cared for, respected, and valued, and usually consists of both practical support (receiving support) and perceived support (31). Actual support includes receiving material assistance and direct services from others; while perceived support refers to an individual’s emotional experience of being respected, understood, and supported (32). Given its predictive function of individual psychological distress and psychological resilience, perceived social support plays a key role in maintaining an individual’s level of mental health (33). Death anxiety, a pervasive and numerous potentially harmful psychological disorder, has also been shown to be moderated by an individual’s ability to perceive social support (34).

An individual’s sense of security consists of two dimensions: external security and internal security (35). External security involves an assessment of the perceived safety and stability of the environment, including public order, interpersonal relationships, welfare systems, and resources (36–38); internal security is the individual’s subjective feeling of being physically and psychologically free from threat (39). In addition, the sense of control and psychological consistency also affect the individual’s perception of his or her safety, and enhance the confidence in coping with stress and difficulties (40, 41). It has been found that the sense of security is an important indicator and manifestation of mental health, and when an individual’s external or internal sense of security is impaired, he or she is prone to mental health problems such as severe stress, depression, anxiety, and post-traumatic stress disorder (42–44). Ottu et al. investigated and found that after experiencing a life-threatening security event, those who value their lives based on a higher quality of life are more sensitive to the threat to their sense of security and controllability in their lives and more likely to manifest their feelings of security and control over their lives. Threatened were more sensitive and more likely to show significant anxiety about death (45). In addition, Scheffold et al. have argued that people who are insecure in their interpersonal relationships exhibit more severe death anxiety due to trust barriers or lack of appreciation of others’ willingness to assist (46).

Despite the links that have been established between these psychological factors and death anxiety, the majority of existing studies have adopted a variable-centered approach, which focuses on analyzing the linear relationship between death anxiety and a single variable, such as finding that social support is negatively associated with death anxiety and that levels of self-esteem buffer anxiety (47), rather than exploring individual differences in the manifestations of death anxiety (48). However, such approaches struggle to capture the heterogeneity of youth populations, ignoring the fact that different individuals may develop differentiated patterns of anxiety through a combination of psychological profiles (e.g., high self-esteem but low security, low social support but high sense of meaning) (49). This neglect of individual differences makes existing intervention strategies for youth death anxiety often lacking in relevance.

Furthermore, while existing studies have provided valuable insights into death anxiety in specific groups such as older adults and healthcare professionals, there remains a significant gap in understanding the heterogeneity of death anxiety among young adults in Mainland China. This gap limits the development of targeted psychological interventions. Theoretically, integrating Terror Management Theory with person-centered approaches like Latent Profile Analysis can elucidate how different psychological buffers (e.g., self-esteem, perceived social support, security) interact within subgroups to mitigate death anxiety. Practically, identifying these distinct profiles can inform more precise and effective mental health support strategies for youth facing existential challenges.

Therefore, the present study aimed to identify heterogeneous patterns of death anxiety among Chinese young adults using latent profile analysis, and to examine the predictive roles of self-esteem, perceived social support, and security. By adopting a person-centered approach, this study sought to provide a more nuanced understanding of how different psychological resources function as protective factors against death anxiety, thereby offering evidence to inform youth-targeted interventions and death education strategies.

Based on the above rationale, we proposed the following hypotheses:

H1: Death anxiety among young adults exhibits heterogeneity and can be empirically classified into distinct latent profiles.
H2: Higher levels of self-esteem, perceived social support, and security are negatively associated with death anxiety and predict membership in lower-anxiety profiles.
H3: Among these psychological resources, security has the strongest protective effect against death anxiety.
H4: Demographic variables such as older age (30–35 years) and recent recovery from acute illness are positively associated with membership in higher death anxiety profiles.

## 2 Methods

### 2.1 Study design and setting

This project is a cross-sectional study and a convenience sampling method was used. All materials for this study were collected from November 2023 to May 2024 through questionnaires distributed in community health and hygiene centers and community residents’ activity centers in Shenzhen and Shaoguan.

### 2.2 Participants

To reduce regional heterogeneity and health-related confounding, we restricted recruitment to community-dwelling young adults in mainland China who were in stable physical and mental health. Detailed eligibility criteria are listed below:

#### 2.2.1 Inclusion criteria

1. Young adults aged between 18 and 35 years;
2. Informed consent and voluntary participation in this study;
3. Given the differences in culture, policy, and economy, participants in this study were limited to residents of mainland China (excluding Taiwan, Hong Kong, and Macao).

#### 2.2.2 Exclusion criteria

1. Those who were unable to cooperate with the survey due to abnormal intelligence or thinking, which could not be assessed by the Simple Intellectual Mental State Scale (total score<24) (50);
2. Those suffering from serious diseases or vital organ dysfunction, such as malignant tumors, heart, liver, and kidney dysfunction;
3. Suffering from diagnosed serious mental illness (e.g., major depression, schizophrenia, etc.) (51, 52);
4. Those who have text reading disorder and cannot understand the content of the questionnaires.

#### 2.2.3 Recruitment and sampling

In this survey, the convenience sampling method was implemented in several community sites in Shenzhen and Shaoguan. We approached a diverse set of individuals within the target age range (different occupations, education levels, and health statuses). Confidentiality and informed consent were emphasized to promote participation and data quality; the achieved sample exceeded the minimum *a priori* requirement.

### 2.3 Sample size calculation

The sample size for this study was determined using multiple methods, and the largest estimated sample size was selected as the primary reference, adjusted for an expected 10% invalid response rate.

#### 2.3.1 Estimation based on mean and standard deviation of death anxiety

Based on previous studies using the Chinese version of the Templer Death Anxiety Scale (C-T-DAS) (53), the mean Death Anxiety score among Chinese university students was 7.83 ± 2.90 (54). With a 95% confidence level (*Z* = 1.96) and an allowable margin of error of 0.3 points, the required sample size was calculated as follows (55):


$$n=\frac{\left(Z_{\alpha /2}^{2}\times \sigma^{2}\right)}{\delta^{2}}=\frac{\left(1.96^{2}\times 2.90^{2}\right)}{0.3^{2}}\approx 359$$

#### 2.3.2 Sample size requirements for latent profile analysis

According to previous simulation studies, conducting an LPA generally requires at least 50–70 participants per identified latent profile, recommending a total sample size typically between 300 and 500 (56). Conservatively estimating up to 5 profiles yields a recommended maximum sample size of 500.

#### 2.3.3 Sample size requirements for multinomial logistic regression

An *a priori* power analysis for multinomial logistic regression was also conducted using G*Power 3.1.9.7 (57). Assuming a medium-to-large effect size (*F²* = 0.25), *α* = 0.05, *power* = 0.80, and including 8 predictors, the minimum required sample size was calculated to be approximately 248.

Considering the highest calculated sample size (n = 500, from LPA requirements) and adjusting for an anticipated 10% invalid response rate, the final target sample size was set as follows:


$$n_{final}=\frac{500}{1-0.10}»556$$

Based on the calculated minimum sample size of 556, we successfully recruited 623 participants, which represents a 12% increase over the required minimum. This over-recruitment was strategic to account for potential data loss or non-response and to better represent the diverse population of young adults in the study area. Specifically, our recruitment efforts were focused on ensuring a wide range of participants in terms of age, gender, occupation, and health status. This approach not only enhanced the statistical power of our analysis but also improved the generalizability of our findings to the broader population of young adults in mainland China. The recruitment process was carefully monitored to ensure that participants were representative of the target population, and the final sample size was approved by the Ethics Committee of the Seventh Clinical Medical College of Guangzhou University of Traditional Chinese Medicine (No. KY-2024-026-01).

### 2.4 Measures

#### 2.4.1 General information questionnaire

Basic information about the participants was collected through a self-developed demographic scale, including gender, age, race, education, occupation, income level, health status, religious beliefs, and life education experiences. Religiosity was measured with a single, binary item (yes/no), a choice dictated by the modest and heterogeneous distribution of religious affiliations in our sample. We acknowledge that this coarse operationalization may fail to adequately capture the nuanced landscape of religious and spiritual beliefs within the Chinese context.

#### 2.4.2 Death anxiety scale

The Templer Death Anxiety Scale (T-DAS) is a self-reported psychometric instrument used by participants (58). It was originally developed by Templer in 1970 and later translated into Chinese and culturally adapted by Chinese scholar Yang Hong in 2003, resulting in the Chinese version of the Templer Death Anxiety Scale (53). The scale consists of 15 items, which can be categorized into four dimensions, including I. Effect (which expresses the individual’s emotions towards experiences and/or views related to death), items: 1, 3, 5, 10, 13, 14; II. Stress and distress (manifesting the individual’s feelings of stress and distress related to death), questions: 4, 6, 9, 11; III. Time Awareness (demonstrating the individual’s attitude toward time and the passage of life), questions 8, 12; and IV. Cognition (which expresses the individual’s perception of survival and death), items 2, 7, and 15. The instrument consists of nine positively scored questions, with a “true” choice indicating a one-point increase; and six negatively scored questions (items 2, 3, 5, 6, 7, and 15), with a “false” choice indicating a one-point increase. The scale has a total score of 15, with 7 being the standard threshold for high death anxiety. The Chinese version (C-T-DAS) has demonstrated good reliability and validity in previous Chinese studies (59). The Cronbach’s alpha for this instrument in this study was 0.726.

#### 2.4.3 Self-esteem scale

The Self-Esteem Scale (SES) was developed by Rosenberg in 1965 (60). In this study, we used the translated and culturally validated Chinese version, which was adapted by Ji Yifu and Yu Xin in 1999 (61). The scale consists of 10 items, including five positively and five negatively worded items. Although Rosenberg supported the use of positive scoring for topics 1, 2, 4, 6, and 7; and reverse scoring for items 3, 5, 8, 9, and 10. However, based on the study of the available information, we insisted that this scale is a single-dimension measurement tool. Besides, Tian, after fully considering the adaptability of the questions in the Chinese context, proposed that maintaining the forward scoring would enable item 8 to obtain better detection results (62). This instrument utilizes a Likert 4-point scale ranging from 1 (strongly disagree) to 4 (strongly agree). The total score is 10-40, with higher scores representing higher levels of overall self-esteem. The Chinese version has established reliability and validity in prior research with Chinese samples (63). In this study, the Cronbach’s alpha (α) for this instrument was 0.850.

#### 2.4.4 Perceived social support scale

The Perceived Social Support Scale (PSSS) was designed by Zimet et al. in 1988 (64). In this project, we used the Chinese version of the PSSS translated by Jiang Qinjin in 2001 to adapt it for Chinese cultural context (65). This scale has 12 items, including three dimensions: family support (items: 3, 4, 8, 11), friend support (items: 6, 7, 9, 12), and support from others (items: 1, 2, 5, 10). The instrument was scored on a 7-point Likert scale with answers ranging from 1 (completely disagree) to 7 (completely agree). The total score ranges between 12 and 84, while the values for a given dimension come from the sum of the corresponding items. The Chinese version has shown good psychometric properties in previous studies (66). In this study, the Cronbach’s alpha (α) for this instrument was 0.905.

#### 2.4.5 Security questionnaire

The Security Questionnaire (SQ) developed by Cong et al. in 2004 was used (67). This instrument was developed specifically for the Chinese population. This instrument contains two dimensions of interpersonal security (questions: 1, 3, 6, 8, 10, 12, 15, 16) and certainty of control (questions: 2, 4, 5, 7, 9, 11, 13, 14), with 16 entries. It is scored on a Likert 5-point scale, with 1–5 representing a scale from Very Compliant to Very Noncompliant. With a total score of 16-80, a high total score is an indication of high security. The original developers reported good reliability and validity (68). The Cronbach’s alpha (α) for this instrument in this study was 0.885.

### 2.5 Procedure

Questionnaires were administered either on site (paper-based questionnaires containing a QR code linking to the electronic form) or online (Questionnaire Star platform). Participants read the information sheet and provided informed consent (signature or ticking “agree”) before proceeding. Surveys were completed individually (10–15 minutes) in private rooms at community centers or via the online platform to ensure privacy. Investigators introduced the study, addressed questions, and monitored emotional comfort where needed. All questionnaires were self-completed anonymously and checked for completeness upon submission.

### 2.6 Statistical analysis

#### 2.6.1 Latent profile analysis

Traditional variable-centered techniques assume population homogeneity, yet death anxiety is likely to manifest in distinct patterns among young adults. To capture these unobserved subpopulations within our heterogeneous youth sample, we used Latent Profile Analysis (LPA)—a model-based, person-centered clustering technique that classifies individuals according to their response patterns on the C-T-DAS. This cross-sectional design with LPA yields qualitatively distinct death-anxiety profiles that variable-centered methods overlook and can inform targeted psychological interventions. Model selection was guided by standard fit indices, including AIC, BIC, adjusted BIC, entropy, and the Lo–Mendell–Rubin likelihood ratio test (LMR-LRT) (69). The latent classes derived from this analysis were subsequently used in regression models to examine the predictive roles of self-esteem and security, as detailed in the following sections.

#### 2.6.2 Data analysis procedures and model selection criteria

First, all received data were independently organized by two researchers using Excel to eliminate duplicates, incomplete information, and responses from foreigners. In the second step, descriptive analysis of the survey data, and reliability analysis of the psychometric instruments were completed using SPSS 27.0. In the third step, Mplus8.3 was prioritized for exploring the latent features of the death anxiety in the youth population, given its hard-to-ignore accuracy in achieving LPA (70). The final class solution was determined by several fitting indices, including the Akaike Information Criterion (AIC), the Bayesian Information Criterion (BIC), and the Adjusted Bayesian Information Criterion (aBIC). When a model possesses the lowest AIC, BIC, and aBIC values indicate the best-fitting model (71). Lo-Mendell-Rubin (LMR) and Bootstrap-Likelihood-Ratio-Test (BLRT) possess the trait of *P* < 0.05 can help determine the superiority of the K class model compared to the K-1 class model (72). The closer the entropy is to 1, the higher level of classification accuracy the model possesses, and Entropy > 0.80 was considered acceptable (73). Moreover, the proportion of members of each profile (the category with the lowest content >5%) (74) and the values on the diagonal in the average attribution probability matrix (higher values indicate a higher level of categorization correctness, and >0.7 is an acceptable result) (75) are also taken into account sufficiently before obtaining a decision on the final solution. In addition to this, the simplicity and interpretability of the theory and results are considered before the final decision on the profile. In the fourth step, concerning the characteristics of the profiles obtained above, we estimated the predictive effect of socio-demographic characteristics on death anxiety profile shifts through descriptive analysis and multivariate logistic regression (76, 77). Finally, using one-way ANOVA and multiple *post hoc* tests (Bonferroni Correction) (78), We assessed the relationship of specific profiles with self-esteem, perceived social support, and security. Any doubts encountered during the questionnaires screening process were resolved by reviewing the raw data and through two-person consultations. In this project, statistical tests were conducted using two-sided tests, and differences were considered statistically significant at *P<* 0.05.

### 2.7 Data quality control

To minimize bias and ensure data quality, multiple control measures were implemented throughout the study. During the design phase, a pilot study was conducted to assess the clarity and feasibility of questionnaire items. All research staff received standardized training on survey administration, emphasizing ethical communication and emotional sensitivity due to the nature of the topic. During data collection, participants were clearly informed about the confidentiality and voluntariness of the study. Questionnaires were checked for completeness upon submission, and participants’ emotional responses were monitored and managed as needed. For data processing, a double-entry procedure was employed to reduce transcription errors, and responses that did not meet predefined exclusion criteria were removed prior to analysis.

### 2.8 Ethical considerations

This project was approved by the Ethics Committee of the Seventh Clinical Medical College of Guangzhou University of Traditional Chinese Medicine (No. KY-2024-026-01), and we strictly adhered to the Helsinki Declaration and its amendments throughout the study. Prior to data collection, trained investigators explained the study purpose and procedures to participants, and informed consent was obtained by signing a written consent form included on the first page of the questionnaire. Participation was entirely voluntary, with no academic or financial incentives provided, and participants were free to withdraw at any time without consequence. Ethically related questions could be addressed directly to the research team in person or via email. All data were anonymized and stored securely on an encrypted institutional server, with access restricted to authorized members of the research team. Paper-based questionnaires were sealed in opaque envelopes and stored in locked cabinets, while electronic data were saved in password-protected folders maintained by the two project managers. In accordance with institutional data management policies, all anonymized data will be retained for five years and securely deleted thereafter. The data will be used solely for the purposes of this research and will not be shared with third parties, ensuring strict confidentiality and compliance with ethical standards.

## 3 Results

### 3.1 Characteristics of participants

In this survey, a total of 704 questionnaires were returned. We excluded over-age, duplication, incomplete information, overseas responses, and responses with a completion time of< 120 seconds. With an effective response rate of 88.5%, we finally obtained 623 reliable evidences. Participants had a mean age of 23.62 years (*SD* = 3.61, *range* = 18–35). Of the participants, 429 were women (68.9%); the majority were Han Chinese (95.7%); a total of 341 (54.7%) self-reported being healthy; and less than a quarter had received life education (23.8%). More detailed information is available in **Table 1**.

**Table 1:** Demographic characteristics of participants.

Characteristics	Total (N=623)
Gender (n,%)	
Male	194 (31.1%)
Female	429 (68.9%)
Age (n,%)	
18-23	341 (54.7%)
24-29	234 (37.6%)
30-35	48 (7.7%)
Ethnicity (n,%)	
Han Nationality	569 (95.7%)
Minority Nationality	27 (4.3%)
Education Attainment (n,%)	
Secondary school	2 (0.3%)
Junior High School	10 (1.6%)
High School or Secondary Vocational School	30 (4.8%)
Junior College or University	471 (75.6%)
Postgraduate Student	110 (17.7%)
Career (n,%)	
Students	293 (47%)
Service Sector	66 (10.6%)
Manufacturing Industry	37 (5.9%)
Office Clerk	116 (18.6%)
High-Tech Industry	87 (14%)
Unemployed	24 (3.9%)
Annual Income (n,%)	
Not yet employed, and no wages	297 (47.7%)
Less than 50,000 yuan/year	63 (10.1%)
50,000-100,000 yuan/year	167 (26.8%)
100-200,000 yuan/year	78 (12.5%)
More than 200,000 yuan/year	18 (2.9%)
Health Status (n,%)	
Healthy	341 (54.7%)
Sub-Healthy	206 (33.1%)
Chronic Non-Communicable Diseases	60 (9.6%)
Rehabilitation from Acute Illness	16 (2.6%)
Religion (n,%)	
Yes	65 (10.4%)
No	558 (89.6%)
Death Education Experience(n,%)	
Yes	148 (23.8%)
No	475 (76.2%)
DAS (mean, SD)	9.9 (3.041)
SES (mean, SD)	27.86 (5.086)
PSSS (mean, SD)	53.49 (12.777)
SQ (mean, SD)	47.29 (11.314)

SD, Standard Deviation; DAS, Death Anxiety Scale; SES, Self-Esteem scale; PSSS, Perceived Social Support Scale; SQ, Security Questionnaire.

Health status was self-reported and categorized as: Healthy = no self-reported health problems; Sub-healthy = minor discomfort without diagnosed chronic disease; Chronic Non-Communicable Diseases = diagnosed long-term non-communicable disease (e.g., hypertension, diabetes); Rehabilitation from Acute Illness = recovering from acute illness

### 3.2 Underlying profiles and characteristics of death anxiety

To obtain the best results, we selected 1–6 latent feature models for exploratory latent feature analysis of death anxiety, and their fitting quality is shown in **Table 2**. In models with 1 to 6 categories, AIC and BIC values decreased as the category increased. However, although the 4-class model performed best on the AIC/BIC metric, considering the higher Entropy (*Entropy* = .962) and stronger category interpretability, we finally selected the 3-CLASS model (see **Table 2**). The model divided the participants into: The High Death Anxiety Group (Profile 1,56.2%) scored the highest on all death anxiety dimensions, especially on the ‘stress and pain’ dimension, which was much higher than the other two groups; The moderate cognitive low death anxiety group (Profile 2,8.8%) had higher cognition of death, but the lowest level of anxiety; the low cognitive moderate death anxiety group (Profile 3,35%) had the lowest score in the dimension of ‘Cognition of life and Death’, however, the overall anxiety level was moderate. Furthermore, in Model results were reliable (see **Table 3** for details).

**Table 2:** Fit statistics for latent profile analysis models.

NO. of profiles	AIC	BIC	aBIC	Entropy	LMRT	BLRT	n (%) per profile
1	7207.694	7243.171	7217.772	——	——	——	623 (100%)
2	6818.347	6875.996	6834.723	0.962	<. 001	<. 001	273 (43.8%)/350 (56.2%)
3	6591.476	6671.298	6614.151	0.962	.031	<. 001	350 (56.2%)/55 (8.8%)/218 (35%)
4	6531.048	6633.043	6560.210	0.880	.011	<.001	68 (10.9%)/55 (8.8%)/218 (35%)/282 (45.3%)
5	6509.370	6633.537	6544.641	0.892	——	——	55 (9%)/218 (35%)/69 (11%)/14 (2%)/267 (43%)
6	6503.403	6649.743	6544.973	0.852	.538	.040	26 (4.2%)/36 (5.8%)/307 (49.3%)/141 (22.6%)/ 91 (14.6%)/22 (3.5%)

AIC, Akaike information criterion; BIC, Bayesian information criterion; aBIC, adjusted Bayesian information criterion;LMRT, Lo–Mendell–Rubin adjusted likelihood ratio test; BLRT, bootstrap likelihood ratio test. p values are reported as<.05,<.01, or<.001 as appropriate.

**Table 3:** Classification accuracy of latent profiles.

Potential Profile	1	2	3
1	1.000	0.000	0.000
2	0.000	0.961	0.039
3	0.006	0.019	0.974

Diagonal values represent the average posterior probabilities of individuals classified into each profile (classification accuracy). Values of.000 indicate probabilities<.001.

**Figure 1:**
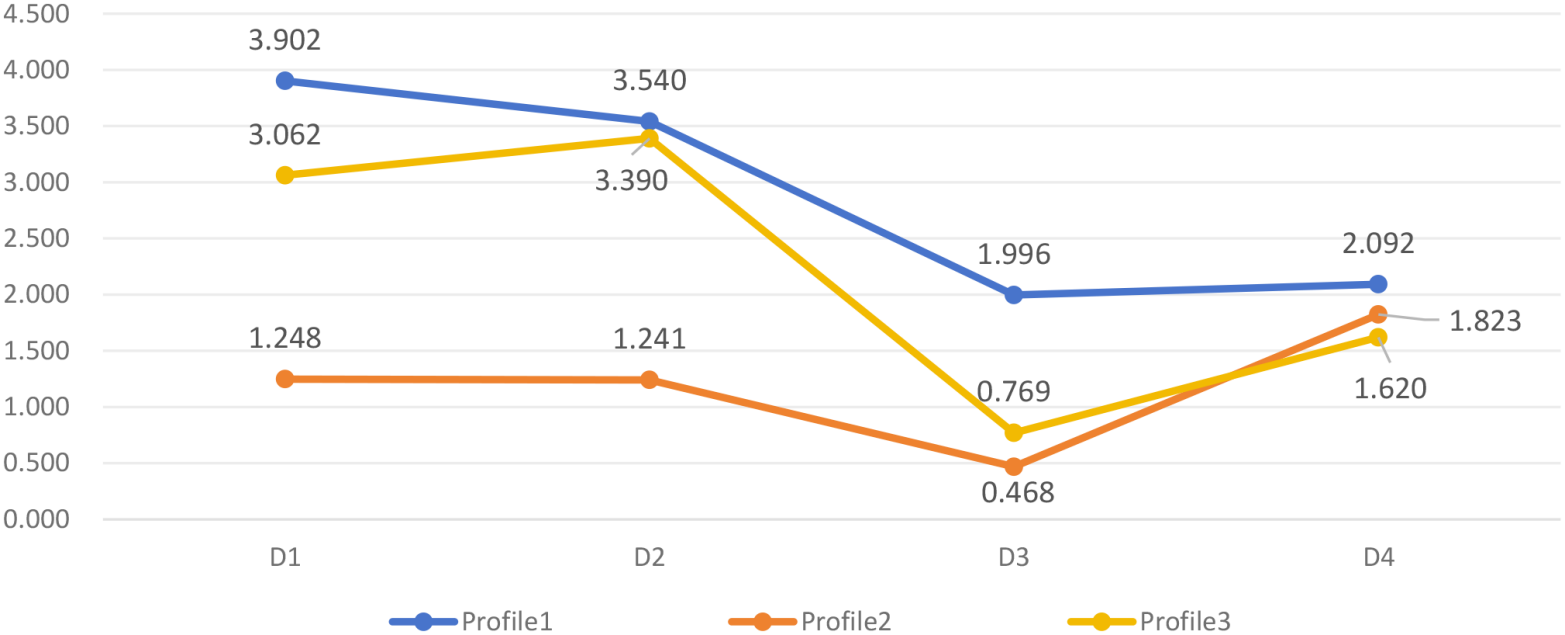
Mean scores of death anxiety dimensions across the three latent profiles. D1, Emotional; D2, Stress and Pain; D3, Time Awareness; D4, Cognition; Profile1, High Death Anxiety; Profile2, Moderate Cognition and Low Death Anxiety; Profile3, Low Cognition and Moderate Death Anxiety.

Based on the results of the potential profile analysis, the researchers plotted **Figure 1**. Detailed statistical values are provided in **Table 4**. Profile 1, which included 56.2% of the participants (N = 350), was characterized by significantly above-average total scores and significant performance excellence in all four death anxiety themes. Hence the name “High Death Anxiety”. Profile 2 is the smallest, consisting of 55 members representing 8.8 percent of the total. People assigned to this group had moderate but low death anxiety scores on the” Awareness of life and death” scale. Therefore, this profile was labeled “ Moderate Cognition and Low Death Anxiety”. Profile 3 comprised 35% (218 persons). This profile scored moderate on the death anxiety scale, but it scored lowest on the” Awareness of life and death” dimension. Hence the name” Low Cognition and Moderate Death Anxiety”.

**Table 4:** Mean scores of death anxiety dimensions across latent profiles.

Death Anxiety Dimensions	Profile 1 (n=350) M±SD	Profile 2 (n=55) M±SD	Profile 3 (n=218) M±SD
Emotional	3.90±1.491	1.24±1.170	3.06±1.594
Stress and Pain	3.54±0.631	1.16±0.631	3.39±0.629
Time Awareness	2.00±0.000	0.47±0.504	0.77±0.424
Cognition	2.09±1.006	1.85±0.970	1.62±0.963

SD, Standard Deviation; Profile1, High Death Anxiety; Profile2, Moderate Cognition and Low Death Anxiety; Profile3, Low Cognition and Moderate Death Anxiety.

An SD of 0.000 indicates identical responses across participants in Profile 1 for this dimension.

### 3.3 The demographic characteristics of each profile

Through descriptive analysis and one-way ANOVA, we characterized the sociodemographic characteristics of each profile. The” Low Cognition and Moderate Death Anxiety” group comprised more than half of the participants > 23 years of age, while the proportion of presenters< 24 years of age in the” Moderate Cognition and Low Death Anxiety” group reached 61.8%. More than half of the people in the” High Death Anxiety” group were unemployed and unpaid. In addition, we also found that people in good health were also likely to have higher Death Anxiety and to be in the “Moderate Cognition and Low Death Anxiety” group. See **Table 5** for details.

**Table 5:** Demographic characteristics of participants across latent profiles.

Variables	Profile1 n (%)	Profile2 n (%)	Profile3 n (%)	X^2^/F	P
Gender				6.958	.031^*^
Male	110 (31.4%)	25 (45.5%)	59 (27.1%)		
Female	240 (68.6%)	30 (54.5%)	159 (72.9%)		
Age				12.358	.014^*^
18-23	203 (58.0%)	34 (61.8%)	104 (47.7%)		
24-29	129 (36.9%)	15 (27.3%)	90 (41.3%)		
30-35	18 (5.1%)	6 (10.9%)	24 (11.0%)		
Ethnicity				2.806	.075
Han Nationality	334 (95.4%)	55 (100%)	207 (95.0%)		
Minority Nationality	16 (4.6%)	0 (0.0%)	11 (5.0%)		
Education Attainment				11.602	.515
Secondary school	1 (0.3%)	0 (0.0%)	1 (0.5%)		
Junior High School	4 (1.1%)	1 (1.8%)	5 (2.3%)		
High School or Secondary Vocational School	18 (5.1%)	1 (1.8%)	11 (5.0%)		
Junior College or University	270 (77.1%)	49 (89.1%)	152 (69.7%)		
Postgraduate Student	57 (16.3%)	4 (7.3%)	49 (22.5%)		
Career				51.522	<. 001^*^
Students	184 (52.6%)	19 (34.5%)	90 (41.3%)		
Service Sector	28 (8.0%)	18 (32.7%)	20 (9.2%)		
Manufacturing Industry	13 (3.7%)	3 (5.5%)	21 (9.6%)		
Office Clerk	73 (20.9%)	8 (14.5%)	35 (16.1%)		
High-Tech Industry	39 (11.1%)	6 (10.9%)	42 (19.3%)		
Unemployed	13 (3.7%)	1 (1.8%)	10 (4.6%)		
Annual Income				25.374	.002^*^
Not yet employed, and no wages	183 (52.3%)	18 (32.7%)	96 (44.0%)		
Less than 50,000 yuan/year	40 (11.4%)	5 (9.1%)	18 (8.3%)		
50,000-100,000 yuan/year	81 (23.1%)	26 (47.3%)	60 (27.5%)		
100-200,000 yuan/year	37 (10.6%)	3 (5.5%)	38 (17.4%)		
More than 200,000 yuan/year	9 (2.6%)	3 (5.5%)	6 (2.8%)		
Health Status				43.974	<. 001^*^
Healthy	198 (56.6%)	26 (47.3%)	117 (53.7%)		
Sub-Healthy	111 (31.7%)	15 (27.3%)	80 (36.7%)		
Chronic Non-Communicable Diseases	40 (11.4%)	13 (23.6%)	7 (3.2%)		
Rehabilitation from Acute Illness	1 (0.3%)	1 (1.8%)	14 (6.4%)		
Religion				.426	.808
Yes	35 (10.0%)	5 (9.1%)	25 (11.5%)		
No	315 (90.0%)	50 (90.9%)	193 (88.5%)		
Death Education Experience				3.077	.215
Yes	77 (22.0%)	18 (32.7%)	53 (24.3%)		
No	273 (78.0%)	37 (67.3%)	165 (75.7%)		

p values are from χ² tests (categorical variables) or one-way ANOVA (continuous variables). Health status: Healthy = no health problems; Sub-healthy = minor discomfort without chronic disease; Chronic Non-Communicable Diseases = diagnosed long-term non-communicable disease (e.g., hypertension, diabetes); Rehabilitation from Acute Illness = recovering from acute illness. *, p<.05*.

### 3.4 Analysis of predictive factors for death anxiety profiles in Chinese youth

To identify predictors of profile membership, we incorporated sex, age, occupation, income level, and health status into multinomial logistic regressions based on observations of demographic characteristics, and we examined which demographic variables predicted membership in each death anxiety profile, the” High Death Anxiety” group was used as a reference.

In profile 2, participants aged 24–29 years reported lower death anxiety compared to those aged 30–35 years. In profile 3, participants aged 18–23 and 24–29 years reported lower death anxiety than those aged 30–35 years, and participants with Acute Illnesses in the Rehabilitation Period showed significantly higher anxiety than those with other health conditions. See **Table 6** for details.

**Table 6:** Multinomial logistic regression predicting profile membership.

Variables (reference items)	B	SE	OR	95%CI	P
Profile2 (reference: Profile1)					
Gender (reference: Female)					
Male	0.079	0.348	1.082	0.547-2.140	.821
Age (reference:30-35)					
18-23	-0.862	0.623	0.422	0.125-1.433	.167
24-29	-1.264	0.606	0.282	0.086-0.926	.037
Career (reference: Unemployed)					
Students	0.335	1.101	1.398	0.162-12.084	.761
Service Sector	1.660	1.194	5.259	0.507-54.571	.164
Manufacturing Industry	0.694	1.323	2.002	0.150-26.780	.600
Office Clerk	-0.237	1.220	0.789	0.072-8.621	.846
High-Tech Industry	0.387	1.234	1.472	0.131-16.525	.754
Salary Level (reference: More than 200,000 yuan/year)					
Not yet employed, and no wages	-0.953	1.034	0.392	0.052-2.978	.366
Less than 50,000 yuan/year	-1.085	0.903	0.338	0.058-1.984	.230
50,000-100,000 yuan/year	-0.228	0.791	0.796	0.169-3.753	.773
100-200,000 yuan/year	-1.520	0.937	0.219	.035-1.372	.105
Health Status (reference: Rehabilitation Period For Acute Illnesses)					
Healthy	-1.667	1.496	0.189	0.010-3.544	.265
Sub-Healthy	-1.710	1.508	0.181	0.009-3.479	.257
Chronic Non-Communicable Diseases	-1.247	1.518	0.287	0.015-5.627	.411
Profile3 (reference: Profile1)					
Gender (reference: Female)					
Male	-0.410	0.211	0.664	0.439-1.005	.053
Age (reference:30-35)					
18-23	-1.119	0.399	0.327	0.149-0.714	.005
24-29	-0.779	0.371	0.459	0.222-0.949	.036
Career (reference: Unemployed)					
Students	-0.547	0.462	0.579	0.234-1.431	.237
Service Sector	-0.008	0.619	0.992	0.295-3.342	.990
Manufacturing Industry	0.233	0.669	1.262	0.340-4.685	.728
Office Clerk	-0.640	0.574	0.528	0.171-1.625	.265
High-Tech Industry	0.055	0.586	1.056	0.335-3.334	.926
Salary Level (reference: More than 200,000 yuan/year)					
Not yet employed, and no wages	0.653	0.705	1.922	0.483-7.648	.354
Less than 50,000 yuan/year	0.037	0.651	1.038	0.290-3.719	.955
50,000-100,000 yuan/year	0.673	0.600	1.959	0.604-6.354	.262
100-200,000 yuan/year	0.501	0.611	1.650	0.498-5.468	.413
Health Status (reference: Rehabilitation Period For Acute Illnesses)					
Healthy	-3.068	1.081	0.047	0.006-0.387	.005
Sub-Healthy	-2.875	1.087	0.056	0.007-0.475	.008
Chronic Non-Communicable Diseases	-4.299	1.151	0.014	0.001-0.130	<. 001*

SE, standard error; CI, Confidence Interval. Profile1, High Death Anxiety; Profile2, Moderate Cognition and Low Death Anxiety; Profile3, Low Cognition and Moderate Death Anxiety. *, p<.05*.

### 3.5 Comparison of various potential profiles of death anxiety considering self-esteem, perceived social support, and security

Analysis of variance showed that the death anxiety group had significant differences in self-esteem (*F =* 5.500, *P = .*004), social support (*F =* 4.163, *P = .*016), and security (*F =* 50.717, *P* <.001)(see **Table 6**). Bonferroni *post hoc* comparison further revealed that the High Death Anxiety Group -Profile file 1) had significantly lower self-esteem scores than the Medium Cognitive Low Death Anxiety group (Profile 2) and Low Cognitive Medium Death Anxiety group (Profile 3)(*P* <. 001). The social support score of the high death anxiety group was lower than that of the other two groups (*P =*. 004), but significant only in friend support and other support dimensions (*P* <. 001). For the security, the score of Profile 2 was the highest, which was significantly higher than that of the other two groups (*P* <. 001). Specific details of these comparisons can be found in **Table 7**.

**Table 7:** ANOVA and post Hoc tests comparing psychological variables across profiles.

Variables	Profile1 (n=350) M±SD	Profile2 (n=55) M±SD	Profile3 (n=218) M±SD	F	P	Bonferroni
Self-Esteem	27.28±4.843	28.22±5.616	28.70±5.223	5.500	.004*	1 < 2 1 < 3^*^ 2 < 3
Perceived Social Support Scale	52.27±13.017	53.58±13.927	55.44±11.874	4.163	.016*	1 < 2 1 < 3^*^ 2 < 3
Family Support	17.36±5.184	17.20±5.612	18.15±5.086	1.762	.173	1 > 2 1 < 3 2 < 3
Friends Support	18.59±5.035	19.35±5.085	19.69±4.541	3.538	.030*	1 < 2 1 < 3^*^ 2 < 3
Others Support	16.32±4.960	17.04±4.996	17.60±4.646	4.673	.010*	1 < 2 1 < 3^*^ 2 < 3
Security Questionnaire	43.69±9.879	55.40±11.068	51.02±11.308	50.717	<. 001*	1 < 2^*^ 1 < 3^*^ 2 > 3^*^
Sense of Interpersonal Security	22.97±5.831	27.47±5.928	26.30±6.125	28.553	<. 001*	1 < 2^*^ 1 < 3^*^ 2 > 3

M, mean; SD, Standard Deviation. Profile1, High Death Anxiety; Profile2, Moderate Cognition and Low Death Anxiety; Profile3, Low Cognition and Moderate Death Anxiety. *, p<.05*.

## 4 Discussion

This study employed latent profile analysis (LPA) to delineate distinct death-anxiety profiles among Chinese youth and to explore the role of self-esteem, social support, and security in the different profiles. The main findings indicated that the youth group showed significant heterogeneity in death anxiety and could be categorized into three latent profile groups: high death anxiety group (56.2%), medium cognitive low death anxiety group (8.8%), and low cognitive medium death anxiety group (35%). Significant differences in demographic characteristics (e.g., age, occupation, health status) and psychological variables (self-esteem, social support, and security) were found among the different groups, validating the research hypotheses.

1. Heterogeneity of Youth Death Anxiety and its Psychological Buffering Mechanism

In this study, we used LPA for the first time to identify the type of death anxiety in a youth group, which breaks through the limitations of the traditional variable-centered approach and reveals inter-individual variability. The three identified profiles reflect distinct psychological coping patterns: Profile 1 (High Death Anxiety, 56.2%) was characterized by low levels of self-esteem, social support, and security, consistent with the “anxiety-buffer disruption” perspective of Terror Management Theory; Profile 2 (Moderate Cognition and Low Death Anxiety, 8.8%) showed the highest security levels, aligning with attachment theory; Profile 3 (Low Cognition and Moderate Death Anxiety, 35%) indicated limited cognitive engagement with death yet elevated emotional distress, suggesting an avoidance-based strategy. These patterns demonstrate that different constellations of psychological resources lead to qualitatively distinct patterns of death anxiety, underscoring the need for targeted interventions. Reviewing the available data, two profiles of death anxiety called the low death anxiety group and the high stress group were identified in a survey of oncology nurses by Chen et al. (79). Although the populations of interest varied, our study also identified two profiles with similar presentations, which were “Moderate Cognition and Low Death Anxiety” and “Low Cognition and Moderate Death Anxiety”. In addition, we also identified “High Death Anxiety” and it was significantly represented among young people (56.2%), which indicates that more than half of young people have severe death anxiety. We suggest that this may be closely related to the failure of the anxiety-buffer system caused by the instability of the current cultural worldview of Chinese young people (e.g., lack of death education) (80). Supporting this explanation, Duan et al. developed the “Peaceful Tea House” program, which provided a safe environment for participants to discuss death-related topics and successfully reduced death anxiety (81).

The present study found variability in death anxiety among young individuals, supporting the hypothesis of heterogeneity in death anxiety. Notably, this variability may stem from the fact that individuals employ different psychological mechanisms to cope with the threat of death. According to Terror Management Theory, individuals buffer their anxiety through cultural worldviews, pursuing self-esteem, and making social connections when faced with the threat of death (82). When these buffers function well, death anxiety remains manageable; when disrupted by excessive anxiety, individuals intensify defensive efforts such as bolstering cultural worldviews, pursuing self-esteem, or strengthening close relationships (83, 84). Thus, young people with low death anxiety can adequately regulate negative emotions about death through a well-functioning anxiety buffer system and do not exhibit an overreliance on the cultural worldview buffer. However, once this buffer system is disrupted by severe death anxiety and causes a disruption in the functioning of the individual’s anxiety-buffering system. They will have to increase their use of holistic defenses, such as working hard to attain some cultural representations, maintaining self-esteem and intimacy, and warding off the damage of death anxiety by gaining the experience of immortality beyond death (34) (85, 86). This seems to explain the phenomenon that participants in Profile 2 had neither severe death anxiety nor outstanding performance in self-esteem and perceived social support compared to Profiles 1 and 3, while those included in Profile 3 showed higher levels of self-esteem and perceived social support compared to Profile 1. This result is consistent with previous research where Kheibari et al. helped participants who experienced death reminders to reduce death anxiety through self-esteem inflation, and those who achieved higher self-esteem showed more pro-social intentions and death anxieties (87). At the same time, Dürst et al. found that social support derived from interactions with relatives helped hospitalized critically ill patients experiencing visitation bans during the COVID period to improve their illness experience and reduce their stress-related symptoms, general anxiety, and death anxiety with family members (88).

The negative correlation between feelings of safety and death anxiety reveals a core psychological mechanism by which humans cope with existential fears. Safety is the state of being free from violence, harm, or other threats to individuals and society, and encompasses not only the protection of life and physical integrity, but also the safeguarding of property, economic interests, lifestyles, and psychological stability (89). From the perspective of Terror Management Theory, security is closely linked to the stability of an individual’s cultural worldview, which may contribute to the perception of death as a natural life process rather than an uncontrollable threat (90). As a result, high-security individuals are more inclined to buffer death anxiety by reinforcing social support and pursuing meaning in life, rather than falling into excessive fear of life’s fragility. Attachment theory further suggests that creating a “secure base” that provides people in need with opportunities to seek help from significant others, to be available and supported, to experience relief and comfort, and to return to other activities can increase their safety and peace of mind, thereby reducing fear and anxiety about death (91). Adequate feelings of safety can help people resist negative emotions as well as relieve anxiety and stress, promoting an individual’s physical and mental well-being, whereas a lack of safety may predict poorer mental health (92). This explains why Profile 2, with the strongest security, exhibited the lowest death anxiety, in contrast to Profiles 1 and 3. Similar findings were found in Mahat-Shamir et al.’s study, whereby individuals’ concerns about their safety significantly increased death anxiety during a sustained wave of terror. Relative to the salience of death accomplished via digital media, those who gained horrific experiences by talking to eyewitnesses had a more moderate expression of death anxiety, which may be a reflection of individuals’ ability to gain interpersonal security in close relationships and interpersonal attachments and to counteract death anxiety (93). In the Chinese context, however, Confucian values of filial piety and pervasive taboos around open discussion of death may erode such secure bases, undermining the cultural worldview that normally buffers existential fears and thereby intensifying death anxiety among youth. Culturally adapted death education programs that respectfully integrate traditional values while providing safe spaces for death-related discourse may thus be essential (94).

In summary, the results align with H1–H4, pointing to heterogeneous manifestations of death anxiety and the likely buffering effects of self-esteem, social support, and security.

2. Differential Effects of Demographic Variables

The present study further revealed the significant predictive role of demographic characteristics on death anxiety subgroups, and we found that age and health status may influence death anxiety among young people. According to Chopik’s inference, as age rises, individuals may gain more socially supportive strengths, and these close relationships can function as emotion regulators to help reduce thoughts and anxiety about death (95). However, this idea was not successfully verified in the data we obtained. Older participants (30–35 years) within the young adult group exhibited higher death anxiety. This trend may stem from their transitional life stage, which involves increasing responsibilities and existential reflections. Young adulthood is an important stage when individuals leave family-centered intimacy and establish personal-centered intimacy, and the relational shifts experienced during this period may significantly alter the trajectory of an individual’s life and affect his or her mental health (96). Unlike young adults who have just reached adulthood, late adolescents to middle-aged individuals may have left their parents’ homes and started their own families with loved ones. While they have a richer social support network, more complex social roles and the pressures that come with those roles are inevitable. This may have influenced their death anxiety (97, 98). In addition to this, young people in poor health, such as those recovering from an acute illness, also reported more severe death anxiety. A sudden acute illness may catch young people off guard with the threat of death, and if they are unable to regulate this emotion within a short period, they may experience a steep increase in death anxiety. This is similar to some clinical observations, as Malinauskaite et al. found that post-traumatic stress symptoms were present in survivors of acute coronary syndromes and that these individuals were significant in terms of fear of death, helplessness, avoidance, and severe anxiety (99). In conclusion, we conclude that older and ex-acute patients among the young should receive more attention, especially when they exhibit symptoms related to death anxiety. Beyond age, other demographic factors such as occupation, income, and gender may also interact with psychological resources to shape death anxiety, though these effects were less pronounced in our data.

### 4.1 Limitation and future perspectives

Although this study provides new insights, there are still some limitations. First, the samples in this study were from two cities in mainland China, and the cultural context may affect the generalizability of the results. The use of convenience sampling and a cross-sectional design further limits the generalizability of these findings to all Chinese youth. In particular, the broad age range of 18–35 years encompassed individuals at different developmental stages, such as students, early-career workers, and those assuming family responsibilities. This heterogeneity may have masked subgroup differences in death anxiety and reduced the precision of our findings. Moreover, although participants came from diverse occupational backgrounds, including healthcare and other death-related professions, subgroup analyses for these high-risk groups were not conducted due to limited sample sizes. This omission may have obscured occupation-specific manifestations of death anxiety.

Furthermore, the cultural specificity of our findings warrants careful consideration. Cross-cultural evidence suggests that while social support serves as a universal buffer against stress, its culturally specific forms, for example esteem-building as opposed to closeness-fostering support, generate divergent emotional outcomes in Western individualist and Eastern collectivist contexts (100). Therefore, the mechanisms linking social support to death anxiety identified in this Chinese sample may not be directly generalizable to other cultures. Future comparative studies are needed to test the cultural generalizability of these findings.

Second, the cross-sectional design of the present study only revealed the correlations between variables and could not infer whether self-esteem, social support, and security were causal factors of death anxiety. Future studies may adopt a longitudinal research design to explore the role of these psychological factors in the development of death anxiety; incorporate qualitative research (e.g., in-depth interviews) to further explore the psychological mechanisms of different anxiety groups; and may test the differences in the effects of interventions (e.g., positive thinking training, supportive groups) on different trait groups to optimize practice protocols.

Third, the self-report questionnaire was used in this study, which may have social desirability bias; future studies may combine behavioral measures or qualitative interviews to obtain more comprehensive data. Additionally, although religiosity was measured, its binary operationalization may not capture the nuanced role of spiritual beliefs in China. Future studies should employ more refined measures to explore the influence of religion on death anxiety. Finally, the failure to include more potential influencing factors (e.g., personality traits, traumatic experiences) may have omitted important explanatory variables, and future studies could incorporate more diverse measures, such as behavioral experiments and physiological indicator tests, to more accurately assess the independent and interactive effects of these factors on death anxiety.

Therefore, these results should be interpreted as exploratory and hypothesis-generating rather than confirmatory, and future studies with probability sampling designs are needed to validate these conclusions.

## 5 Conclusion

This study revealed the heterogeneity of death anxiety in a youth population through LPA, identifying three latent profile clusters: a high death anxiety group (56.2%), a medium cognitive low death anxiety group (8.8%), and a low cognitive moderate death anxiety group (35%). The results indicated that self-esteem, perceived social support, and security were significant negative predictors of death anxiety, supporting the hypothesis of Terror Management Theory. The high death anxiety group had significantly lower self-esteem and social support scores than the other two groups, whereas the group with a higher security showed lower levels of death anxiety. In addition, age and health status had a differential effect on the death anxiety subgroup, with death anxiety being more pronounced in young adults aged 30–35 years and in individuals recovering from acute illness. This study offers a precise foundation for youth mental health interventions, emphasizing the need for tailored strategies to support high-death anxiety groups. However, the study was limited by the cross-sectional design and regional sample, and it is necessary to further validate the causal mechanism by combining longitudinal studies, cross-cultural comparisons, and multivariate methods (e.g., behavioral experiments), as well as exploring the role of potential influences, such as personality traits, in the future.

## Data Availability

The raw data supporting the conclusions of this article will be made available by the authors, without undue reservation.
